# Functional connectivity changes in the entorhinal cortex of taxi drivers

**DOI:** 10.1002/brb3.1022

**Published:** 2018-08-15

**Authors:** Limin Peng, Ling‐Li Zeng, Qiang Liu, Lubin Wang, Jian Qin, Huaze Xu, Hui Shen, Hong Li, Dewen Hu

**Affiliations:** ^1^ College of Mechatronics and Automation National University of Defense Technology Changsha Hunan China; ^2^ Research Centre of Brain Function and Psychological Science Shenzhen University Shenzhen Guangdong China; ^3^ Cognitive and Mental Health Research Center Beijing Institute of Basic Medical Sciences Beijing China

**Keywords:** entorhinal cortex, fMRI, functional connectivity, navigation, resting‐state

## Abstract

**Introduction:**

As a major interface between the hippocampus and the neocortex, the entorhinal cortex (EC) is widely known to play a pivotal role in spatial memory and navigation. Previous studies have suggested that the EC can be divided into the anterior‐lateral (alEC) and the posterior‐medial subregions (pmEC), with the former receiving object‐related information from the perirhinal cortex and the latter receiving scene‐related information from the parahippocampal cortex. However, the functional connectivity maps of the EC subregions in the context of extensive navigation experience remain elusive. In this study, we analyzed the functional connectivity of the EC in subjects with long‐term navigation experience and aimed to find the navigation‐related change in the functional properties of the human EC.

**Methods:**

We investigated the resting‐state functional connectivity changes in the EC subregions by comparing the EC functional connectivity maps of 20 taxi drivers with those of 20 nondriver controls. Furthermore, we examined whether the functional connectivity changes of the EC were related to the number of taxi driving years.

**Results:**

Significantly reduced functional connectivity was found in the taxi drivers between the left pmEC and the right anterior cingulate cortex (ACC), right angular gyrus, and bilateral precuneus as well as some temporal regions, and between the right pmEC and the left inferior temporal gyrus. Notably, the strength of the functional connectivity between the left pmEC and the left precuneus, as well as the right ACC, was negatively correlated with the years of taxi driving.

**Conclusion:**

This is the first study to explore the impact of long‐term navigation experience on the connectivity patterns of the EC, the results of which may shed new light on the potential influence of extensive navigational training on the functional organization of the EC in healthy human brains.

## 1 INTRODUCTION

As is well‐known, driving a car is a complex behavior that involves environmental perception, action execution, and route navigation. When humans navigate, the knowledge about their local environment, which is stored in their memories and helps them find their way to their destination, is indispensable. Many studies have shown that navigation is supported by an ensemble of areas, including the regions related to landmark knowledge (e.g., the parahippocampus) and spatial representation (e.g., the precuneus, cuneus, and inferior parietal lobe), as well as the regions containing place cells, grid cells, and head direction cells (e.g., the hippocampus and retrosplenial cortex) (Epstein, 2008; Maguire et al., 1998; O'Keefe & Nadel, 1979). It is believed that these structures interact to update our current position and plan to find the shortest or most feasible route to achieve the navigational goal (Byrne, Becker, & Burgess, 2007).

The key role of the hippocampus in spatial memory and navigation is well‐established (Maguire et al., 1998), and structural changes in the hippocampus have been found in people with a high dependence on navigational skills (Maguire et al., 2000), which reflects the capability for local plastic change in the structures of the human brain in response to environmental demands. In this region, the entorhinal cortex (EC), as the major interface connecting the neocortex and hippocampus (Muñoz & Insausti, 2005), has received a large amount of attention in recent years. The EC is located on the ventromedial surface of the temporal lobe together with the adjacent perirhinal cortex (PRC) and parahippocampal cortex (PHC), and these regions form a neural circuit that is essential for the formation and retrieval of spatial memory (Brown & Aggleton, 2001; Eichenbaum, Yonelinas, & Ranganath, 2007; Ranganath, 2010; Ranganath & Ritchey, 2012; Suzuki & Howard, 2010). Previous nonhuman studies have observed that the neurons of grid cells in the EC exhibited a hexagonal spatial pattern of spike rates, highlighting the cellular basis of spatial navigation in the EC (Fyhn, Hafting, Witter, Moser, & Moser, 2008; Hafting, Fyhn, Molden, Moser, & Moser, 2005; Sargolini et al., 2006). This specific pattern tiles the entire environment that is available to the animal and has been proposed to represent a neural code for path integration. However, the relationship between the EC and long‐term navigational behavior is still poorly understood, which greatly limits our understanding of how the brain supports navigation.

Early research has shown that activation of the hippocampus is strongly associated with the accurate knowledge of where places are located and with the accurate navigation between them (Maguire et al., 1998); thus, the EC, as an essential part of the hippocampus, plays a crucial role in coordinating object and spatial information from the environment. Recently, the functional topography of the human EC was established using high‐field functional MRI, which divided the EC into the anterior‐lateral (alEC) and the posterior‐medial subregions (pmEC) (Maass, Berron, Libby, Ranganath, & Düzel, 2015; Navarro Schröder, Haak, Zaragoza Jimenez, Beckmann, & Doeller, 2015). These studies revealed that the alEC has particularly strong connections with the PRC, which is involved in the processing of object sources, and that the pmEC is closely connected to the PHC, which is involved in the processing of visual scenes.

Although numerous studies have focused on the distinct functions of the EC subdivisions within human brains, the functional connectivity maps of the EC subregions with substantial navigation experience remain elusive. It has been reported that the EC is involved in the processing and conveying of scene and item information (Reagh & Yassa, 2014; Schultz, Sommer, & Peters, 2012), and we hypothesized that extensive navigation behaviors might lead to a functional change in the EC, which would particularly influence the connectivity patterns of the EC subdivisions. In this study, we analyzed the functional connectivity of the EC in subjects with long‐term navigational experience and aimed to find the navigation‐related change in the functional properties of the human EC. As known, taxi drivers must undergo extensive training, learning how to navigate between thousands of places in a big city, and it is necessary for them to pass a very stringent set of police examinations before they were licensed to operate taxis, including the tests about the traffic transportation theories and road driving skills. Only when they are qualified for all the examinations, they can obtain their taxi driving licenses from the National Road and Traffic Administration. Therefore, taxi drivers are ideally suited for the study of spatial navigation. A number of cross‐sectional studies, which focused on the navigational expertise and used the taxi drivers as a model, confirmed that the taxi drivers performed significantly better than controls on tests assessing knowledge of local landmarks and their spatial relationships (Maguire, Woollett, & Spiers, 2006; Woollett & Maguire, 2009). In this study, choosing the well‐established bilateral alEC and pmEC regions (Maass et al., 2015) as regions of interest (ROI), we examined the functional connectivity changes in the EC subregions by comparing the EC functional connectivity maps in 20 licensed taxi drivers with those of 20 control subjects with no driving experience; the taxi drivers were chosen to ensure a consistent level of driving in the environment.

In our recent works, the functional connectivity strength between the frontal‐parietal network and primary visual network and the average connectivity dynamics within the vigilance network were both observed to exhibit a significant correlation with the number of years the taxi drivers had been driving (Shen et al., 2016; Wang, Liu, Shen, Li, & Hu, 2015), suggesting that the taxi driving years may be an important factor to affect functional patterns in the brains of taxi drivers. In this study, therefore, we also examined whether the functional connectivity changes of the EC were related to the number of years the taxi drivers had been driving.

## 2 MATERIALS AND METHODS

### 2.1 Subjects

The participants of the study were 20 licensed taxi drivers and 20 healthy control subjects (right‐handed) with no driving experience, and the two groups were matched for sex, age, and educational level. These participants were all recruited in Chongqing, China, which is located in a mountainous region with quite a complicated traffic situation. All of the taxi drivers, who worked approximately 8 hr a day, had been driving for an average time of 11.6 years (range 6–23 years), and the average time for them being licensed taxi drivers was 4.9 years (range 1–14 years). The participants in the control group did not know how to drive at all, and their transportation modes consisted of on foot and the bus system. All of the participants had no history of major head trauma or any neurological disorder, and none of them had been addicted to alcohol or drugs. The Institutional Review Board of Southwest University approved the study, and all subjects provided written informed consent in order to participate. More information about the two groups is found in Table 1.

**Table 1:** The characteristics of the participants recruited in this study

Variables	Drivers	Non‐drivers	*p* value
Sample size	20	20	
Age (years)	39.5 ± 5.8	41.1 ± 5.0	0.34^a^
Sex (male/female)	20/0	18/2	0.15^b^
Education (years)	9.5 ± 1.8	9.0 ± 1.4	0.37^a^
Years of taxi driving	4.9 ± 3.5		
Years of total driving	11.7 ± 4.9		

Note

^a^Two‐sample *t* test. ^b^Pearson chi‐square test.

### 2.2 Data acquisition and preprocessing

Each subject was instructed to keep conscious with their eyes closed during the resting‐state scan, not thinking of anything in particular. All of the participants reported that they kept stationary and remained awake for the entirety of the experiment. The resting‐state fMRI data were collected using a Siemens Trio 3‐T MRI scanner in the Key Laboratory of Cognition and Personality (Southwest University), Ministry of Education, China. The imaging parameters were as follows: number of axial slices = 32, TR = 2000 ms, TE = 30 ms, slice thickness = 3.0 mm, flip angle = 90°, FOV = 200 × 200 mm^2^, and in‐plane resolution = 64 × 64. For each subject, the resting‐state scan lasted 8 min, and 240 volumes were obtained.

The preprocessing of the resting‐state fMRI data was carried out using the statistical parametric mapping software package (SPM8, http://www.fil.ion.ucl.ac.uk/spm), as described in our previous studies (Zeng, Shen, Liu, & Hu, 2014a; Zeng et al., 2014b, 2018). The first 10 volumes of each scan were discarded to avoid the magnetic saturation effect. Then, slice‐timing and head motion correction were performed, in which the remaining images were realigned to the first volume within a run for the correction of interscan head motions. All the participants in this study had less than 2 mm translation and 2° of rotation in any of the *x*‐, *y*‐, and *z*‐axes. Next, spatial normalization, spatial smoothing, and temporal filtering were performed, with the images normalized (2 mm isotropic voxels) to the standard EPI template in the Montreal Neurological Institute (MNI) space, spatially smoothed with a Gaussian filter kernel of 4 mm full‐width half‐maximum and temporally filtered with a Chebyshev bandpass filter (0.01–0.08 Hz). Finally, to reduce the spurious variance unlikely to reflect neuronal activity, 18 nuisance signals were removed from the filtered images by multiple regression, including three mean signals from white matter (WM), the cerebrospinal fluid (CSF) and the whole brain and six parameters obtained from head motion correction, as well as their first‐order derivative terms. The residuals of the regression were used for further analysis.

### 2.3 Functional connectivity analysis

**Figure 1:**
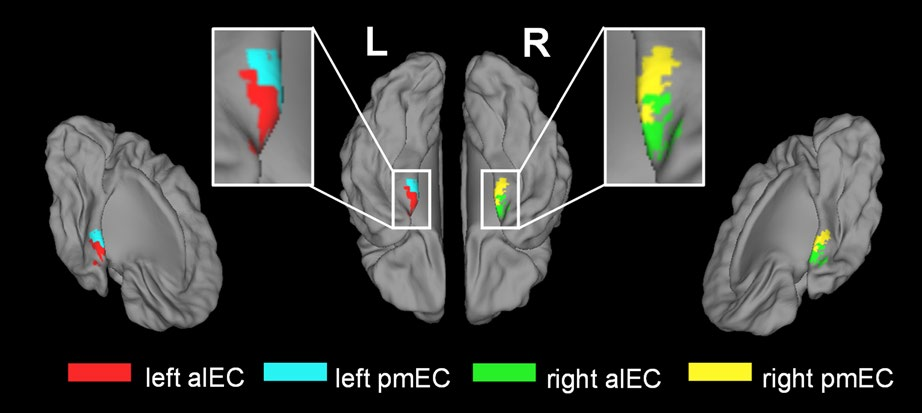
The locations of the ROIs for the left alEC (red), left pmEC (blue), right alEC (green), and right pmEC (yellow)

To examine the changes of the resting‐state EC functional connectivity patterns in the taxi drivers relative to the nondriver controls, a ROI‐based correlation method was used. We chose the previously defined bilateral alEC and pmEC (Maass et al., 2015) as our ROIs (Figure 1), which have been reported to show preferential intrinsic functional connectivity with the PRC and the PHC, respectively. More specifically, in the study by Maass et al. (2015), a whole EC mask was manually defined for each hemisphere on the T1 group template generated from all individual structural images, and then the functional correlations between the voxels in the mask and PRC and PHC ROIs (manually segmented for each subject on the individual high‐resolution structural images) were calculated for each subject. Finally, the voxels were clustered into two subregions across all the participants, that is, alEC and pmEC, according to their PRC and PHC connectivity preference. In this study, the masks of the alEC and pmEC in the MNI space created by Maass and colleagues (available at https://doi.org/10.7554/elife.06426.014) were directly used as the ROIs in the functional connectivity analysis.

The ROI signal was calculated by averaging the time courses over all the voxels within each ROI. For each individual, we obtained the functional correlation maps by calculating the Pearson's correlation coefficients between each ROI signal and the time series of each voxel in the entire brain. Subsequently, Fisher's *z* transformation was applied to the resulting maps to improve the normality.

One‐sample *t* tests (*p *<* *0.001, uncorrected, cluster size ≥30 voxels) were first conducted for the *z*‐maps of each ROI within the taxi driver and control groups, respectively. Then, a spatial *z*‐map mask of a given ROI was obtained by combining the binary spatial maps of the two groups together (selecting the voxels in either binary spatial map). And two‐sample *t* tests were finally performed on the individual *z*‐maps in a voxel wise manner by applying the spatial *z*‐map masks (*p *<* *0.05, AlphaSim corrected). The AlphaSim correction (cluster radius connection: rmm = 5; number of Monte Carlo simulations = 1,000) was performed using the AlphaSim program in REST toolbox (http://www.restfmri.net), which applied Monte Carlo simulation to calculate the probability of false‐positive detection by taking both the individual voxel probability threshold and cluster size into consideration (Chao‐Gan & Yu‐Feng, 2010). A combination of individual voxel's *p *<* *0.01 and cluster size >38, 41, 36, 32 voxels was used to attain the significance of corrected *p *<* *0.05 for left alEC, left pmEC, right alEC, and right pmEC, respectively. Moreover, to further evaluate the effect of the taxi driving years on the variability of the functional connectivity of the EC, the correlation coefficient was calculated between the strengths of the functional connectivity showing significant between‐group differences (*p *<* *0.05, AlphaSim corrected) and the number of years of taxi driving.

### 2.4 Analysis of gray matter volume

Voxel‐based morphometry (VBM) analyses were performed to examine structural changes in the EC subregions. Using the DARTEL toolbox in SPM8, all structural images were first brain extracted, then tissue‐type (i.e., gray matter, WM, and CSF) segmented, nonlinearly registered to each other and normalized to MNI space. On the normalized gray matter images, the gray matter volume (GMV) of each EC subregion was calculated by summing up all the values of voxels within the corresponding mask of each ROI. Two‐sample *t* tests were finally conducted to examine the GMV differences between the two groups for each ROI. In addition, the functional connectivity showing significant between‐group differences would be reanalyzed by including the GMV of the corresponding EC subregion and the GMV of the target region, as regressors in the statistical analysis.

## 3 RESULTS

### 3.1 Functional connectivity of the EC seed regions

**Figure 2:**
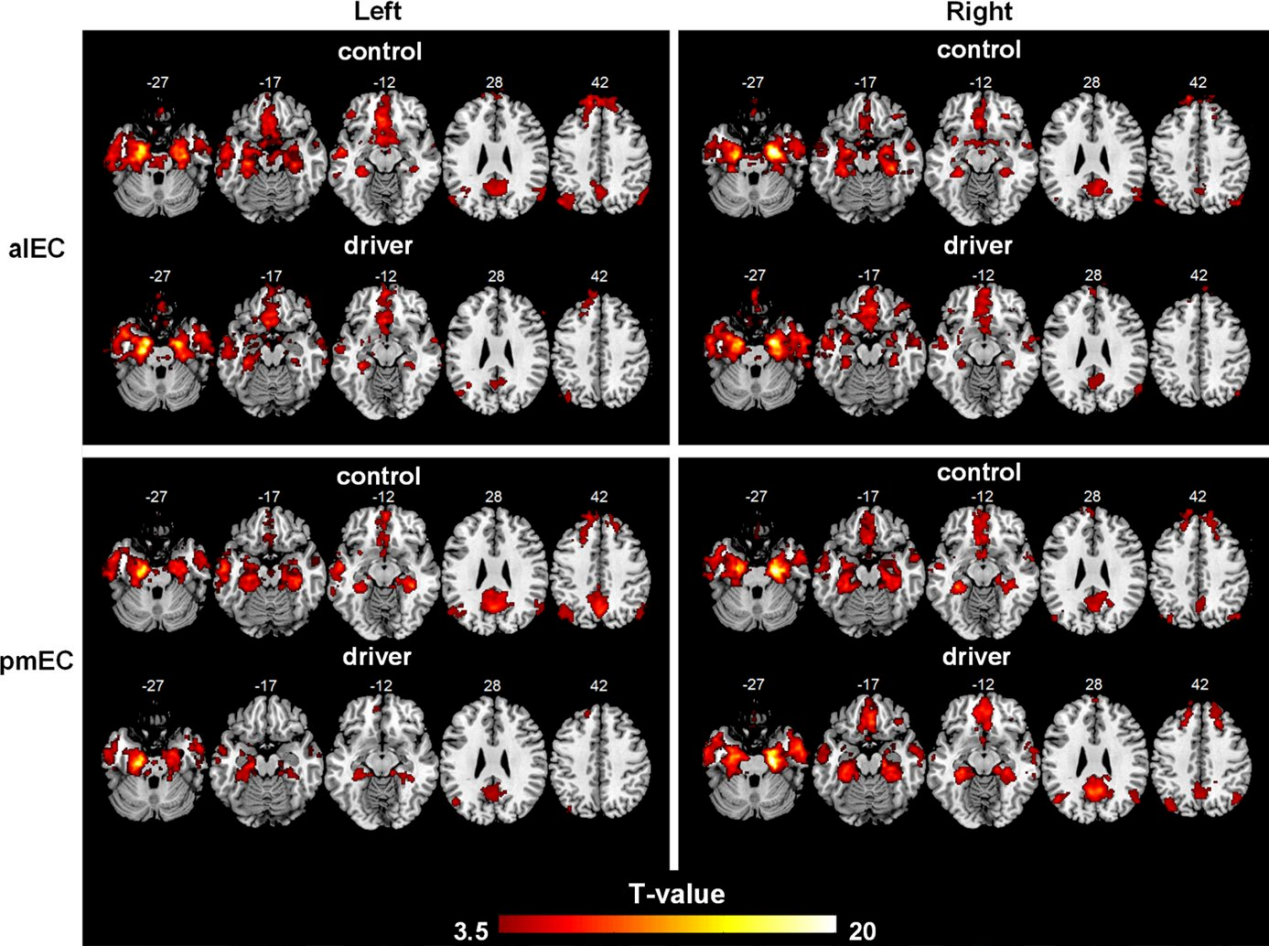
Whole‐brain voxel wise analysis for the functional connectivity of the alEC and the pmEC. One‐sample *t* test results (*p *<* *0.001, uncorrected) are mapped separately for drivers and nondrivers

Figure 2 shows the results of the whole‐brain functional connectivity analysis after the one‐sample *t* tests for the alEC and pmEC in the two groups. As seen, for both the drivers and the nondriver controls, the functional connectivity profiles of the EC were quite similar to each other. For both the alEC and pmEC, strong functional connectivity was found with major regions of the temporal lobe and the parietal lobe, as well as with some frontal regions, including the precuneus, angular gyrus, hippocampus, parahippocampus, and some lateral temporal regions. More specifically, the alEC exhibited more preferential connectivity with the anterior default mode network, primarily including the medial prefrontal cortex and the ACC; while the pmEC exhibited more preferential connectivity with the posterior default mode network, primarily including the posterior cingulate cortex, angular gyrus, and precuneus.

### 3.2 Reduced EC functional connectivity in taxi drivers

**Figure 3:**
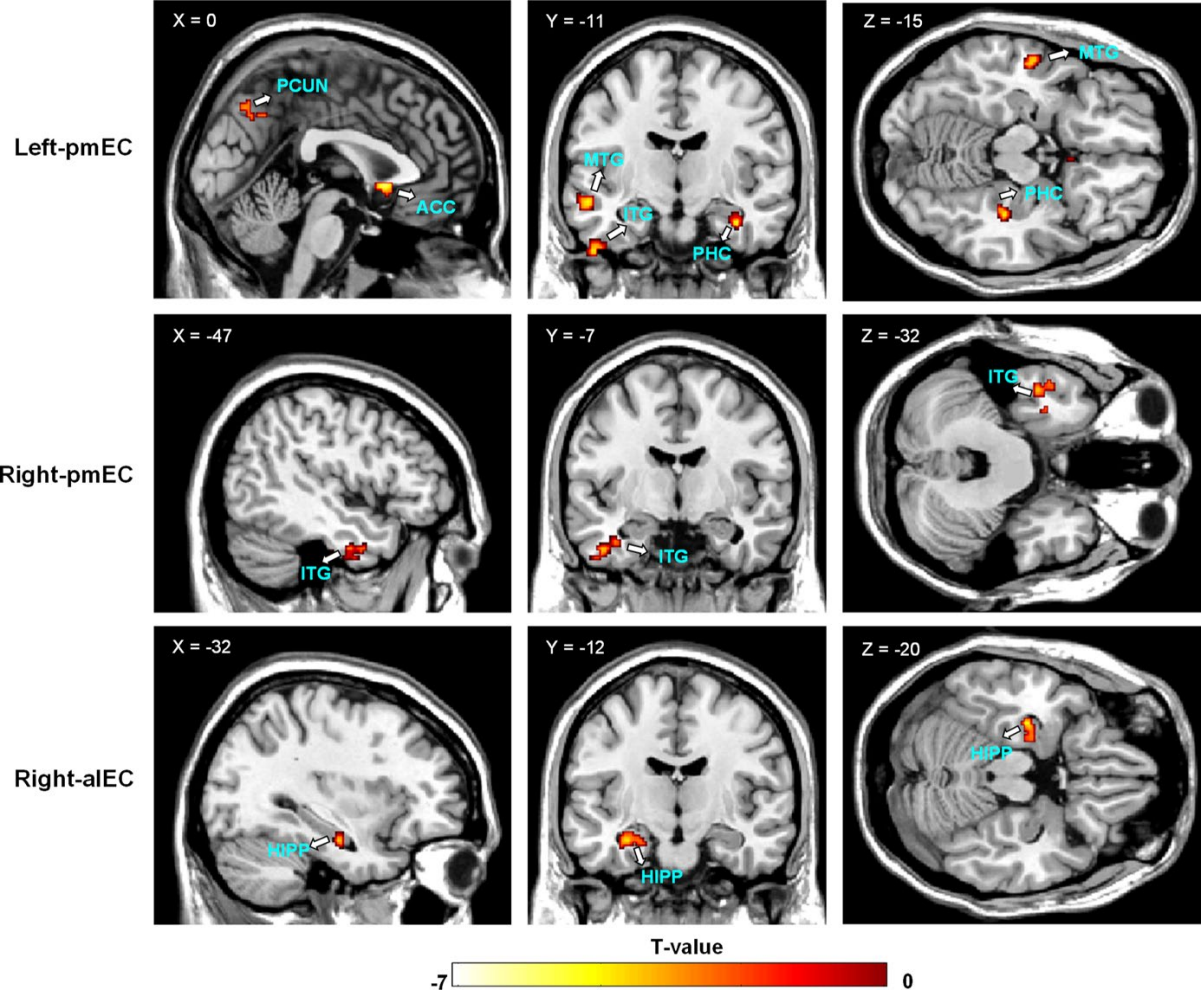
Comparison of the EC connectivity patterns between the drivers and nondrivers. Two‐sample *t* test results (*p *<* *0.05, AlphaSim corrected) are mapped separately for the alEC and pmEC

In spite of few significant differences between the two groups in terms of their one‐sample *t* test results, we found that the taxi drivers showed significantly reduced EC functional connectivity with the DMN relative to the nondriver group through a comparison analysis (Table 2; Figure 3). The results were displayed at the significance level of *p *<* *0.05, AlphaSim corrected. Specifically, for the left pmEC, lower functional connectivity was found to the right ACC, right angular gyrus, bilateral precuneus, right parahippocampal gyrus, and some temporal regions in the taxi drivers than in the nondrivers (cluster size >41 voxels), the majority of which are located in the DMN. The right pmEC showed only decreased functional connectivity in the drivers with the left inferior temporal gyrus (ITG) (cluster size >32 voxels). Moreover, compared with the control subjects, the taxi drivers exhibited decreased functional connectivity between the right alEC and the left hippocampus (cluster size >36 voxels), whereas the functional connectivity of the left alEC showed no significant differences between the two groups (cluster size >38 voxels). Increased functional connectivity related to the bilateral EC subregions was not found in the taxi drivers compared with the controls.

**Table 2:** The taxi drivers exhibited reduced functional connectivity of bilateral EC subregions relative to nondrivers

Target region	Side	BA	Cluster size (voxels)	MNI coordinates (*x*,* y*,* z*)	*T*‐value
Left‐pmEC					
Inferior temporal gyrus	L	20	146	−52, −12, −44	−4.07
Temporal pole	R	38	58	36, 16, −38	−4.05
Parahippocampal gyrus	R	36	112	38, −12, −24	−5.04
Middle temporal gyrus	L	21	75	−54, −12, −12	−4.08
Anterior cingulate gyrus	R	25	47	0, 14, −2	−4.86
Angular gyrus	R	40	70	62, −56, 32	−3.83
Precuneus	L	7	57	−16, −52, 38	−3.71
Precuneus	R	7	53	6, −66, 40	−3.29
Right‐pmEC					
Inferior temporal gyrus	L	20	38	−40, −20, −38	−3.94
Inferior temporal gyrus	L	20	70	−46, −6, −32	−4.09
Right‐alEC					
Hippocampus	L	28	61	−32, −14, −20	−4.77
Left‐alEC					
None					

### 3.3 Correlation analysis

**Figure 4:**
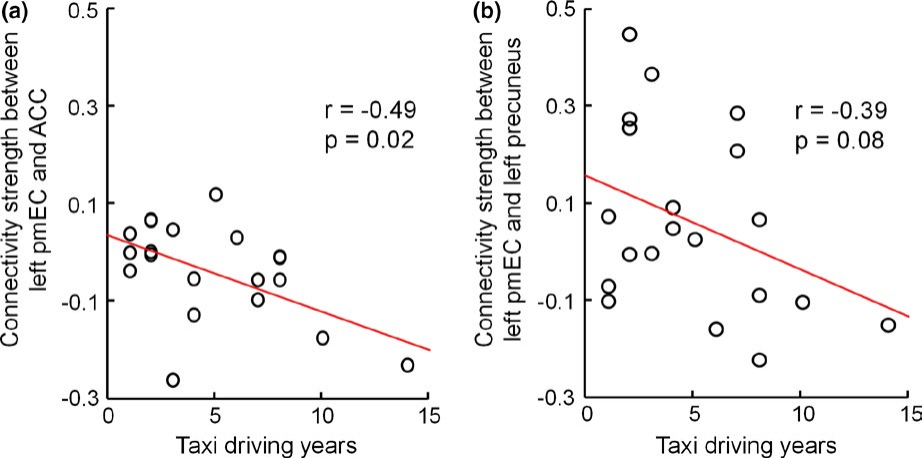
Scatter plots and fitted lines for the number of years of taxi driving against the strength of the functional connectivity between the left pmEC and (a) the right ACC, as well as (b) the right precuneus

To further identify the driving‐related differences between the drivers and the controls, we evaluated the influence of the taxi driving years on the changes in the EC functional connectivity. As illustrated in Figure 4, the functional connectivity between the left pmEC and the right ACC and between the left pmEC and the left precuneus exhibited a negative correlative trend with the number of years of taxi driving (*p *<* *0.1, uncorrected). In addition, the correlation between the altered EC functional connectivity and the number of years of total driving was also evaluated, although no significant correlation was found.

### 3.4 VBM results

To test whether altered functional connectivity of EC subregions in this study might be explained by MRI‐detectable changes of gray matter, VBM analyses were performed on the acquired 3D‐T1 images. But no significant GMV difference was observed in the EC subregions between the taxi drivers and nondriver controls (*p *>* *0.05, uncorrected). Moreover, adding the GMV as covariates in the statistical analyses did not change the functional connectivity results as described in the previous section.

## 4 DISCUSSION

As the core of the brain's navigation system, the precise role of the EC in spatial navigation is still surrounded by much controversy. In the present study, we used resting‐state fMRI analyses to investigate the navigation‐related differences in the connectivity patterns of the EC between taxi drivers with long‐term driving experience and nondrivers. The results revealed changes in the functional connectivity of the EC in the taxi drivers. Specifically, the taxi drivers showed significantly diminished functional connectivity between the left pmEC and the right ACC, right angular gyrus, right parahippocampal gyrus, bilateral precuneus, left middle temporal gyrus, and the left ITG, with the majority of these regions being located in the DMN. Furthermore, markedly reduced functional connectivity was also found in the taxi drivers between the right pmEC and left ITG and between the right alEC and the left hippocampus compared with the control subjects. Notably, in the taxi driver group, the strength of the functional connectivity between the left pmEC and the right ACC, as well as the left precuneus, showed an obvious negative correlation with the number of taxi driving years, which might provide further evidence for the specific changes in the EC functional pattern that results from occupational dependence on spatial navigation. To the best of our knowledge, this is the first study to explore the impact of long‐term driving experience on the connectivity patterns of the EC, the results of which may shed new light on the potential influence of extensive navigational training on the functional organization of the EC in healthy human brains.

In this study, we found diminished pmEC functional connectivity with the DMN in the taxi drivers compared with nondrivers. Previous studies have demonstrated that the DMN constructs an internal model of the spatial layout that integrate input from the PHC and the retrosplenial cortex (RSC) (Ranganath & Ritchey, 2012). Therefore, the lower pmEC functional connectivity to these regions might indicate that the taxi drivers can complete the navigation tasks more independently and efficiently. The taxi drivers were likely to be more familiar with the layout of the city after repeated experiences from driving in the same environment, and the route to the destination might already exist in their minds, which may lead to the lower reliance on the acquirement of temporary spatial information in navigation tasks.

It should be noted that both the left and right pmEC were shown to have significantly reduced functional connectivity with the left ITG. This region is widely considered to be the final stage of the ventral visual processing pathway, receiving its visual input from V2 to V4, and appears to be very crucial for the formation of working memories of visual objects and scenes. On the basis of previous studies, the maintenance of visual information has been reported to be implemented through sustained activation of visual object representations in the ITG, which was further directly correlated with whether the working memories could be successfully converted into long‐term memories (Lee, Simpson, Logothetis, & Rainer, 2005; Rainer, Lee, & Logothetis, 2004; Ranganath, 2006). Our results of the decreased functional connectivity between the pmEC and ITG revealed that the taxi drivers might complete their cognitive tasks with a lower level of visual representation. This finding means that they may rapidly make decisions in complicated natural scenes, even without the visual information being processed by the high‐level visual cortex, and may efficiently form long‐term memories through the current visual image.

More specifically, the left pmEC of the taxi driver group exhibited decreased functional connectivity with the bilateral precuneus, right ACC, and right angular gyrus. In fact, in terms of the type of spatial representation, there exist two classes of spatial coding systems: (a) The allocentric spatial system in which the location of one object is defined relative to the location of other objects; and (b) the egocentric perspective system in which participants locate an object by referring to their own position (Berthoz, 2001; Bohbot et al., 2002; Burgess, Maguire, & O'Keefe, 2002). Additionally, the angular gyrus and precuneus have been reported to exhibit greater activation when the subjects involved the egocentric navigation strategy rather than the allocentric navigation strategy (Boccia, Nemmi, & Guariglia, 2014). Our results showing that the functional connectivity between these areas and the pmEC was diminished in taxi drivers might suggest that a transition with decreased dependence on the egocentric strategy or relatively increased reliance on the allocentric navigation strategy was developed by many of the driving‐related training. These results were consistent with the enhanced need for the competence that allows the taxi drivers to navigate from new starting locations based on the configuration of the environmental cues. In particular, we found that the strength of the functional connectivity between the left pmEC and the left precuneus was negatively correlated with the years of taxi driving. This observation provided further evidence supporting our hypothesis that the functional connectivity changes in the EC were likely to be linked to extensive navigation experience, given that the navigational skills would be gradually enhanced with more driving experience.

It is noted that the aforementioned EC functional connectivity was diminished in the taxi drivers compared with the nondriver controls. Several previous studies showed an increase in functional connectivity between hippocampal and extrahippocampal regions as a function of experience (Aly, Ranganath, & Yonelinas, 2013; Joost & Gabriele, 2011; Sulpizio, Boccia, Guariglia, & Galati, 2016), but there is evidence suggesting that training with lower intensity may lead to enhancement of previously established functional connections, while training with higher intensity may lead to increased efficiency (Karim et al., 2017; Penhune & Steele, 2012). Furthermore, a recent study focusing on the changes in functional connectivity and GABA levels with long‐term motor learning indicated that lower amounts of practice might rely mostly on the established connections by increasing the functional connectivity, whereas higher amounts of practice might cause formation of new connections and result in increased circuit efficiency reflected by decreased functional connectivity (Sampaiobaptista et al., 2015). Thus, the decreased functional connectivity of the EC in the professional taxi drivers observed here may result from high amounts of navigation training.

In the VBM analysis, no significant GMV difference was observed in the EC subregions between the two groups (*p *>* *0.05, uncorrected). Actually, the GMV changes in the target regions with between‐group differences of EC functional connectivity were also examined, and no significant GMV difference was observed either. In addition, GMV did not contribute to the altered functional connectivity patterns observed. Thus, the changes of the functional connectivity of the EC subregions may not be related to the structural differences within the current study sample.

Several potential limitations in this study should be noted. First, the sample size was relatively small, which may limit the statistical power for detecting group differences and the correlation analysis with taxi driving years. In particular, the correlation analysis results were uncorrected, so they should be interpreted with caution. Second, the limitations in spatial resolution made it difficult to acquire BOLD fMRI data at an extraordinary level of anatomical detail. Recent advances in data acquisition methods, such as ultra‐high‐speed and ultra‐high‐field fMRI (Feinberg & Yacoub, 2012), might provide more accurate boundaries of the EC and more reliable statistical characteristics. Furthermore, we could not directly estimate the relationships between the EC functional connectivity and navigational capability due to a lack of quantitative evaluation of the participants' navigational competence. Finally, we used only the resting‐state fMRI of the two groups, but a longitudinal study or a task‐related study should be considered in the future.

## 5 CONCLUSION

This study investigated the navigation‐related functional connectivity changes in the EC in the brains of taxi drivers. We found that the taxi drivers showed significantly reduced functional connectivity between the pmEC and the ITG, PHC, ACC, precuneus, angular gyrus, and middle temporal gyrus, the vast majority of which are located in the DMN. Our results may provide new insights into how extensive navigational training or experience may influence the functional pattern of the EC in the healthy human brain.
